# Super-Resolution Processing of Synchrotron CT Images for Automated Fibre Break Analysis of Unidirectional Composites

**DOI:** 10.3390/polym15092206

**Published:** 2023-05-06

**Authors:** Radmir Karamov, Christian Breite, Stepan V. Lomov, Ivan Sergeichev, Yentl Swolfs

**Affiliations:** 1 The Center Materials Technologies, Skolkovo Institute of Science and Technology, Bolshoy Boulevard 30, bld. 1, 121205 Moscow, Russia; 2 Department of Materials Engineering, KU Leuven Kasteelpark Arenberg 44, 3001 Leuven, Belgium

**Keywords:** fibre breaks, computed tomography, deep learning, super-resolution, image quality

## Abstract

Fibre breaks govern the strength of unidirectional composite materials under tension. The progressive development of fibre breaks is studied using in situ X-ray computed tomography, especially with synchrotron radiation. However, even with synchrotron radiation, the resolution of the time-resolved in situ images is not sufficient for a fully automated analysis of continuous mechanical deformations. We therefore investigate the possibility of increasing the quality of low-resolution in situ scans by means of super-resolution (SR) using 3D deep learning techniques, thus facilitating the subsequent fibre break identification. We trained generative neural networks (GAN) on datasets of high—(0.3 μm) and low-resolution (1.6 μm) statically acquired images. These networks were then applied to a low-resolution (1.1 μm) noisy image of a continuously loaded specimen. The statistical parameters of the fibre breaks used for the comparison are the number of individual breaks and the number of 2-plets and 3-plets per specimen volume. The fully automated process achieves an average accuracy of 82% of manually identified fibre breaks, while the semi-automated one reaches 92%. The developed approach allows the use of faster, low-resolution in situ tomography without losing the quality of the identified physical parameters.

## 1. Introduction

Unidirectional composites (UD) are widely used in industry for their high mechanical properties-to-weight ratio [1]. One of the common damage modes is fibre breakage, which governs longitudinal tensile failure. As fibre breaks accumulate, clusters of fibre breaks will develop, which will eventually lead to catastrophic failure of the UD composite [2]. Accurate prediction of this phenomenon is an important and difficult task that requires not only comprehensive mathematical models but also fast and reliable verification methods. Different prediction models, describing the longitudinal tensile fibre breaks, were introduced in the past decades (for example, [3,4,5,6,7]) and, recently, detailed benchmarking exercises of the models were performed [2,8,9]. The latest benchmark [2] stated that “the models failed to predict fibre break (and cluster) development accurately”. These predictive models displayed a low accuracy for coupled break formation and evolution. The authors of [2] emphasise the importance of an in situ experimental method development in order to further increase the accuracy of longitudinal tensile strength predictions.

Recently, X-ray computed tomography (CT) became the most utilised technique for experimental evaluation of fibre break development during tensile tests [10]. Combined with monitoring in situ performance, CT enables observation of the chronology of damage development. Labscale CT was extensively used to study composite materials, for example, in [11,12,13,14,15,16]. Labscale CT, however, remains too slow to study fibre break development at the representative strain rates and resolutions required for fibre-by-fibre detection level. Therefore, researchers have used synchrotron radiation computed tomography (SRCT), which enables in situ observation of fibre break development over time and during loading [17,18]. However, despite the high cost and complexity of SRCT, we can only study small specimens (around 1 mm^3^) in the targeted high sub-µm resolution. For continuous in situ scanning, the image quality is significantly lower. Owing to the inherent limitations of CT, a 3D image of a specimen at a higher resolution requires increased X-ray flux and exposure time and introduces limitations in the specimen’s size [19]. CT data processing includes the extraction of fibre trajectories, which is usually achieved using automated or semi-automated algorithms. The algorithms work only for high-resolution images (HR), and it is very difficult to annotate the fibre breaks in low-resolution, noisy images.

For low-resolution images (LR), all the identification needs to be performed manually, and several attempts over the same volume are needed to find all the fibre breaks. Considering that in situ scans produce dozens of volumes, and fibre break densities can reach up ~1000 mm^−3^, it can take several working days to identify all the fibre breaks in one volume manually [2]. The resolution limitation of X-ray CT can be one of the many bottlenecks for the rapid development of not only longitudinal tensile failure models but also models for other properties that require information on the damage and microstructure of composite materials.

Generally, there are two methods used to improve the resolution of CT images: hardware- and software-based. When synchrotron radiation is exploited, the best and most expensive hardware is already used, so it is not always possible to upgrade. One of the software-based solutions to address the resolution limitations of tomography is to use image quality enhancement with super-resolution (SR) techniques [20,21,22,23]. In the computer vision field, SR are emerging algorithms designed for improving image quality. In recent years, researchers have made significant progress in reconstructing high-resolution 2D images using deep learning, especially with convolutional neural networks (CNN) and generative adversarial neural networks (GAN) [24,25,26]. For instance, Enhanced Super Resolution GAN (ESRGAN) can achieve excellent enhancement results and demands low computational overhead after training [27]. The main drawback of super-resolution is that the neural networks require a large amount of ideally paired high- and low-resolution images for training, which are difficult to obtain for CT imaging. In addition, if non-synthetic data is used, optical distortions can make it impossible to create a perfectly aligned dataset in 3D due to small differences in feature locations [28]. The problem of data parity can be solved using the CycleGAN architecture [29], as this does not require paired datasets.

Super-resolution techniques are mainly available for 2D images. For 3D images, they can be implemented only in the slice direction, not the in-plane direction [30] or by averaging improvements in the in-plane and slice directions [31]. These 2D approaches can introduce inconsistencies in neighbouring slices and cannot consider the information from the other slices and generate information between them. This is an important drawback for analysing continuous features such as fibres. One of the solutions is to use super-resolution on initial tomography projections before 3D image reconstruction [32], but projections are not always available. Recently, 3D super-resolution algorithms started to appear that use 3D kernels and enable direct 3D image analysis [24,33]. They require more data for training and much more hardware resources. The deeper architecture leads to difficulties in training convergence and requires more advanced strategies to train the model properly.

In this work, we propose the use of deep learning-based super-resolution for CT image quality improvement and, specifically, for enabling automated fibre break analysis in unidirectional composites. To achieve super-resolution of CT images, we developed a deep learning architecture that combines ESRGAN and CycleGAN, which can handle 3D images and generate high-quality results without needing paired data. The study was performed for two types of carbon fibre/epoxy unidirectional composite with already known fibre break distribution [34]. The quality of super-resolution enhancement is evaluated with the following metrics: (1) the number of individual fibre breaks (1-plets), and (2) the number of clusters (2-plets, 3-plets). The implementation of deep learning techniques in the analysis of fibre breaks significantly increases the quality of identification and reduces time and manual intervention requirements.

## 2. Materials and Methods

This study is based on the materials and data obtained by Breite et al. [8] and, recently, by Guo et al. [35]. Here, we provide a brief description of the materials and data acquisition. For more detailed data specifications and access, we refer the readers to the corresponding data article [36,37].

### 2.1. Materials and Manufacturing

Two carbon fibre cross-ply laminates were used: one for training the neural networks and another for validation. Cross-ply laminates are employed to efficiently apply loads to microscale specimens inside the SRCT load rig. The transverse ply did not affect the measured longitudinal fibre breaks.

The first material was produced from prepregs manufactured at KU Leuven in a hot-melt drum winder. The T700SC-12K-50C carbon fibres (Toray Industries) were impregnated with Sicomin SR8500 KTA313 epoxy resin. The composite had a ${\left[90/0\right]}_{s}$ layup.

The second material was made from Grafil 34-700WD-24 K-1.4%A carbon fibres (Mitsubishi Chemical) and proprietary 736LT epoxy resin at North Thin Ply Technology (Switzerland). For this material, a ${\left[90_{4}/0_{4}\right]}_{s}$ layup was produced. Curing of the prepreg took place in KU Leuven’s computerised autoclave according to the manufacturer’s recommendations [38,39].

### 2.2. Synchrotron-Radiation Computed Tomography Experiments

**Figure 1:**
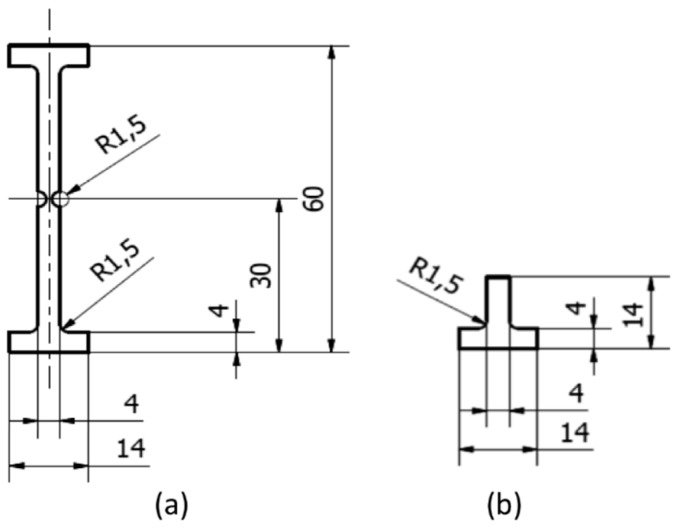
DENT specimen design for SRCT measurements: (**a**) the specimen itself and (**b**) the aluminium end Table All dimensions are in mm. (reprinted from [2] with permission from Elsevier).

After bonding 1 mm thick 2014-T6 aluminium panels in the end tab regions using 3M Scotch-Weld EC 9323 B/A structural adhesive, miniaturised double-edge-notched tensile specimens (DENT specimens) were prepared from the cured materials, using a water-jet cutter, according to the dimensions specified in Figure 1. Bonding end tabs before water-jetting ensured perfect alignment of the end tabs, which is crucial in order to avoid any flexure during the tensile tests.

In total, data was acquired during two separate beamtimes at the TOMCAT beamline at Swiss Light Source (SLS). During the first beamtime, KU Leuven, INSA Lyon, and the University of Southampton performed the SRCT measurements together. INSA Lyon provided the tension-compression rig [40] for the in situ experiments of the NTPT composite (34-700 WD), and the GigaFRoST camera [41] was used for continuous scanning. A second beamtime took place under the participation of Lund University and KU Leuven, in which a customised Deben CT500 tension-compression rig was used. From this beamtime, only static scans without loading are analysed in this study. Here, a pco.EDGE camera was used to acquire high- and low-resolution imaging of the in-house produced material (T700SC)). The high- and low-resolution scans were obtained from one specimen, but, due to mechanical adjustments of the scanning system, small deviations in specimen position and microscope magnification occurred [28].

Table 1 describes all the essential acquisition parameters of the two SRCT datasets.

**Figure 2:**
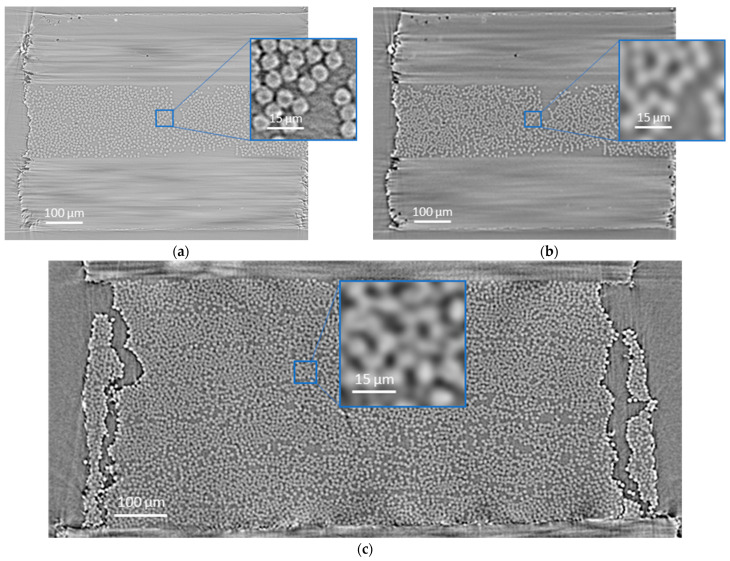
Illustrations of middle slices of the CT scans of: (**a**) T700SC specimen in a high-resolution scan (0.3 µm); (**b**) T700SC specimen in a low-resolution scan (1.2 µm); (**c**) 34-700 specimen in a low-resolution scan (1.1 µm).

Absorption-based tomography reconstruction was performed using the Gridrec algorithm [42] without optical distortion corrections. In total, one high- and one low-resolution volume of the T700SC specimen were prepared to train the neural network. Due to high memory consumption, the CT images were divided into small volumes to create a large training dataset (details in Section 2.4). The low-resolution image was interpolated to have a scale factor of 4 with the HR image, allowing the correct upscaling of 2^2^. In addition, the T700SC LR scan was adjusted to match the 34-700 LR scans in terms of average grayscale values, contrast, and sharpness. Out of 17 low-resolution scans of the 34-700 specimen with fibre break development under continuous load, four images were prepared for fibre break analysis and verification of the super-resolution algorithm: initial (0% load, where 100% load indicates failure of the specimen), intermediate load (75% load), high load (94% load), and before failure (98% load). Figure 2 shows the prepared images, illustrating the difference in sharpness and contrast between high- and low-resolution images.

### 2.3. Super-Resolution Algorithm

In this work, a generative adversarial network (GAN [43]) was designed, trained, and used as a super-resolution algorithm. Traditional GANs comprise two networks, a generator, and a discriminator. In this study, the generator’s objective was to increase image resolution, i.e., to produce a generated high-resolution image from an original low-resolution image. The purpose of the discriminator is to take the original and the generated high-resolution images as input, distinguish the original from generated ones, and pass the feedback on to the generator. In this adversarial process, the generator wants to fool the discriminator, and the discriminator wants to know when and how it is fooled. Both neural networks give better and better results as the training progresses.

**Figure 3:**
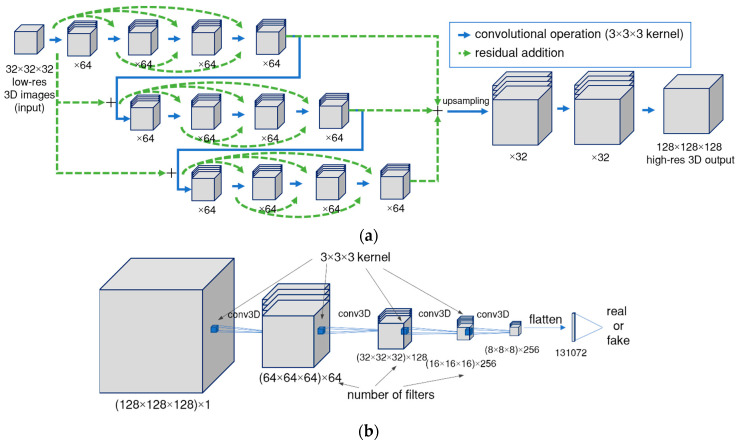
Detailed graphical representation of neural network architectures: (**a**) generator and (**b**) discriminator.

The generator and discriminator are convolutional neural networks with modifications described below. The Enhanced Super-Resolution generator ([27]) with residual-in-residual blocks was implemented and modified to work with grayscale 3D CT images. The generator was also upgraded to the 3D case by employing volumetric kernels. Residual blocks force the information in the initial image to be used throughout all layers of the neural network and allow the neural network to remember the initial image up to the final high-resolution image generation. Figure 3 presents detailed descriptions of the generator and discriminator. As was discussed in the literature [27], the super-resolution method can enhance the image quality when: (1) there is enough data to train the model (this depends on each case and should be tested separately) and (2) the minimal feature to be increased is visible in low-resolution images (for CT images, the damage size should be at least twice the spatial resolution).

**Figure 4:**
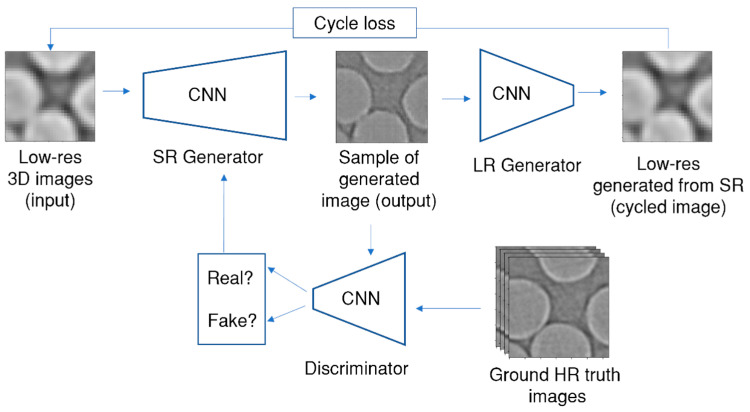
Cycle Generative Adversarial Network for super-resolution of CT images of composite materials.

The network was enhanced with the CycleGAN methodology [29], which allows the use of unpaired images for training; the LR and HR data do not need to be aligned pixel-by-pixel and do not need to depict the same image at all, just a similar one. CycleGANs work by enforcing an inverse transformation; they translate a low-resolution image to look like a high-resolution image without paired constraints during the training. The use of such a GAN mitigates any inconsistencies caused by optical distortions, as the network can operate on unpaired data. The final network architecture has over 8 million trainable parameters and is shown in Figure 4. The following loss functions were used to train the generator and discriminator: reconstruction, adversarial, and cycle losses.

### 2.4. Data Processing and Neural Network Training

Python 3.8 and TensorFlow 2.9 frameworks were utilised to process the CT data, implement the deep learning architecture, and train models. The learning process of neural networks was performed on a workstation with a 12-Core Xeon 4214 processor, a 16 GB Tesla V100 graphical card, and 64 GB of RAM.

Because deep learning and CT processing are hardware-intensive tasks, CT volumes were divided into smaller volumes as follows: 32 × 32 × 32 pixel^3^ for low-resolution images and 128 × 128 × 128 pixel^3^ for high- and super-resolution images. We used a volume overlap of 3 pixels for low-resolution and 12 pixels for high-resolution images. The total training data set consisted of 4560 LR and HR small volumes.

ADAM stochastic gradient descent solver [44] was applied for the model optimisation with $\beta_{1}=0.9$. The mean square error (MSE) loss function was implemented as a cycle loss for pixel-wise comparison between LR and cycled LR images for LR generators. RaGAN (Relativistic average GAN) loss function was used for the generator and the discriminator adversarial training process [45]. The total loss function was the weighted sum of all losses with the weight of cycle loss equal to 10 and GAN losses equal to 1, as in [29]. The training was performed with mixed precision [46].

Low-resolution volumes were enhanced by super-resolution and full-scale SR images were obtained. The overlap allowed us to perform the enhancement almost seamlessly.

### 2.5. Fibre Break Identification

The reference data for fibre break locations were obtained by several manual inspections of images for fibre break analysis, which were performed in detail on the last volume before failure [2,36]. For the specimen with 17 volumes to be analysed, it can take 3–5 working days to identify all the fibre breaks once everything is set up. These difficulties are related to the high fibre break density, which can reach 1000 breaks/mm^3^.

For this paper, all darker regions are called voids, including possible fibre breaks and cracks. For image segmentation, three segmentation algorithms were analysed: RootPainter, ImageJ with Weka segmentation plugin, and InSegt. A modified RootPainter algorithm [47] was implemented for fibre and void cross-section identification as the algorithm with the best performance and fast segmentation speed. Weka segmentation [48] showed similar segmentation results but at a much slower speed. The InSegt algorithm [49] and the classical ImageJ segmentation are only applicable to fibres and do not work well to segment voids.

The RootPainter algorithm is based on deep learning techniques and implements a U-Net network for image segmentation with a batch size of 4 and 3 × 3 kernels. The network was trained according to the RootPainter documentation. The training was performed interactively on partially annotated images, and later on corrective annotations, until the algorithm produced satisfactory results. To locate fibre centre points, only the central parts of the fibres without edges were used for training. Part of the InSegt Fibre code [49] was implemented for fibre trajectory tracking. All voids were analysed in 3D with the MATLAB “regionprops3” function and then filtered to remove noise (voids less than 1000 pixels in volume) and very large objects (more than 10^5^ pixels in volume), which were analysed manually.

Two methods were used to distinguish the fibre breaks from the voids. The first method searched for fibre sections above and below a void along the smallest diameter of a fitted ellipsoid; the voids located between the fibre sections are considered to be fibre breaks. The second method required a CT image of the initial state of the specimen. The resolution of the initial state image was also increased with super-resolution, and a MATLAB image registration algorithm was implemented to align the initial state image with the loaded state images. The voids intersected by the initial fibre trajectories were considered fibre breaks, as was the case in [2], for higher-quality CT scans of stepwise loaded specimens. In the second method, the distance from the centre of the void to the fibre sections was considered to decide on the intersection, in order to avoid false identification due to image artefacts.

The clustering of fibre breaks was analysed according to geometric criteria based on stress redistribution caused by a fibre break. In line with [2], two fibre breaks are considered to belong to the same cluster if their centre points are located in the same cylindrical volume with a radius of 13 µm (2 fibre diameters) and an axial length of 97.5 µm (15 fibre diameters).

The accuracy of fibre break identification was analysed using statistical classification [50]. In this study, we labelled an identification as a true positive when correctly indicating the presence of a fibre break, a false negative when incorrectly indicating the absence of a fibre break, and a false positive when incorrectly indicating the presence of a fibre break. The accuracy defines how close the automated algorithm gets to the number of fibre breaks that were manually identified. Accuracy was calculated using the following equation:

(1) $$Accuracy=\frac{true\;positive}{true\;positive+false\;negative+false\;positive}$$

For semi-automatic identification, the fibre breaks manually identified from large-fused objects are also taken into account and false positives are checked. The miss rate indicates the percentage of fibre breaks that were not found by the automated algorithms.

(2) $$Miss\;rate=1-\frac{true\;positive}{true\;positive+false\;negative}$$

## 3. Results and Discussions

### 3.1. Image Processing

**Figure 5:**
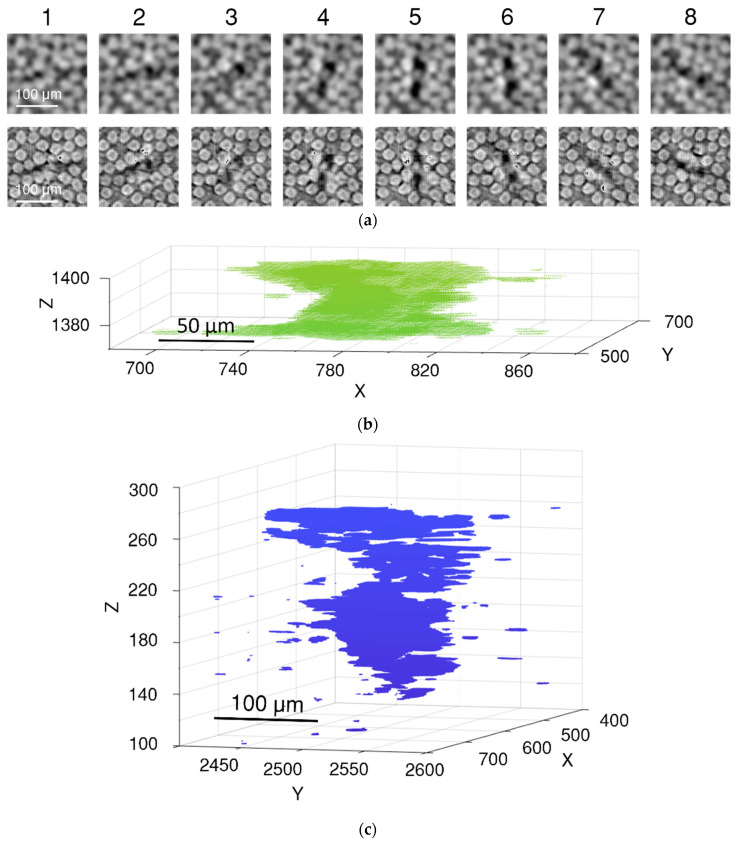
Low-resolution in situ scans have vortex artefacts around the large voids: (**a**) slice-by-slice representation of the artefact; (**b**) 3D visualisation of segmented void; (**c**) visualisation of a large-fused object with surrounding noise.

SRCT images of a continuously loaded (in situ) specimen were analysed as a validation set. Compared to statically acquired images, in situ images not only have a lower resolution but also higher noise and less precise edges of the objects due to movement during image acquisition. For example, voids in the in situ image are not represented as spheres or ellipsoids, but as vortex artefacts with the void as their centre (see Figure 5). This creates additional difficulties for image processing.

**Figure 6:**
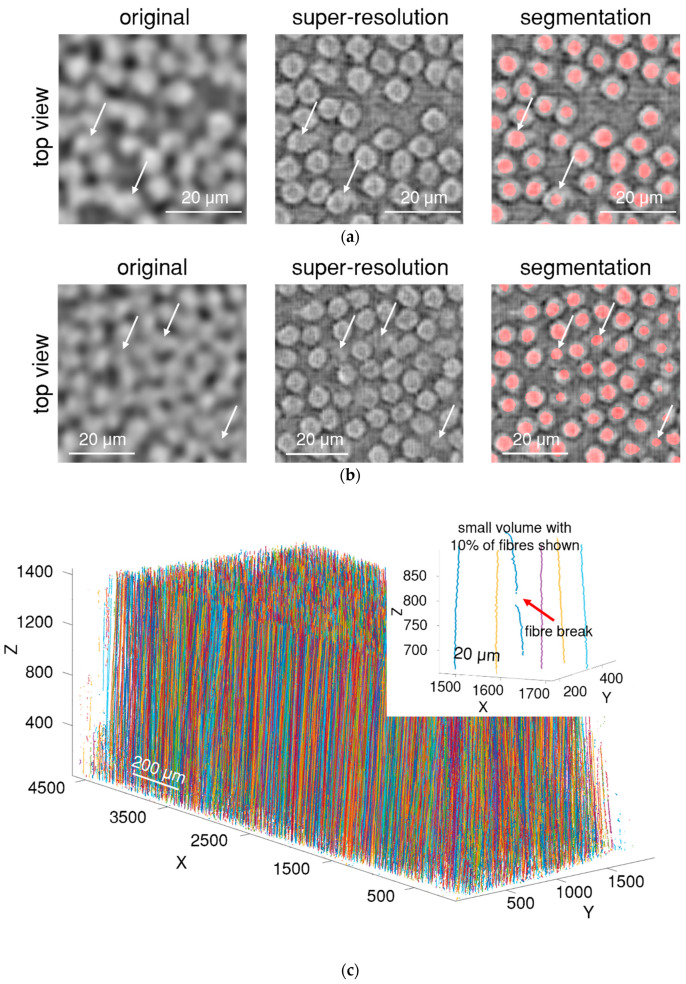
Super-resolution enhancement of fibres and their segmentation in: (**a**) common locations; (**b**) challenging locations; (**c**) visualisation of fibre trajectories (a random colour is assigned to each fibre); in the inset, only 10% of the fibres are shown to demonstrate a fibre break.

Super-resolution algorithms were applied to the in situ synchrotron images. Figure 6 presents the enhanced images. The resolution of the image was increased from 1.1 µm/pixel to about 0.3 µm/pixel; the size of LR images was increased from 1059 × 448 × 1665 px^3^ (1.4 GB) to SR images of 4236 × 1792 × 6660 px^3^ (90 GB). A significant improvement in image quality can be observed: fibre cross-sections become more visible, the fibre edges become defined enough to be visually and computationally distinguished, and fibres do not blend into each other anymore. Stitching artefacts can be found upon careful inspection of the centre of the image in Figure 6a. However, the stitching quality is good and does not affect the image analysis. Overall, the image becomes more suitable for automatic processing. As we do not have ground truth HR images of 34-700 material to compare with SR images, we cannot use image-related metrics (such as peak signal-to-noise ratio) for quantitative assessment.

Despite the significant quality improvement, deep learning deviations are present: (1) several fibres are not round anymore, (2) in rare cases, the edges of the fibres can seamlessly merge into the matrix (white arrow on Figure 6a), and (3) in the most challenging cases, where all the fibres are merged in the original image, a few reconstructed fibres can be not easily detected by the human eye (Figure 6b). However, when deep learning (U-Net) segmentation is used, all the fibres are correctly segmented; the model reliably identifies fibres even in difficult cases where fibres are represented by small greyscale gradients.

The quality of the reconstruction is consistent along the volume in all directions. There are no large variations in the quality enhancement, such as the position of fibre edges or greyscale inconsistencies. This is achieved by using a 3D filter in the super-resolution model and seamlessly stitching small batches into the whole volume.

Fibre segmentation by the U-Net neural network allows fibres to be identified reliably on each slice and to be tracked along the volume. Figure 6c presents the result of the fibre tracking, where each fibre is represented as a line with a random colour. Fibre segmentation and tracking are essential because finding and identifying fibre breaks correctly is impossible without accurate fibre tracking.

The majority (more than 90%) of the fibres are tracked along the entire volume without interruption or loss of tracking. This suggests that super-resolution and deep learning segmentation can be used to enable automatic fibre tracking in low-resolution CT images of fibre-reinforced composite materials.

**Figure 7:**
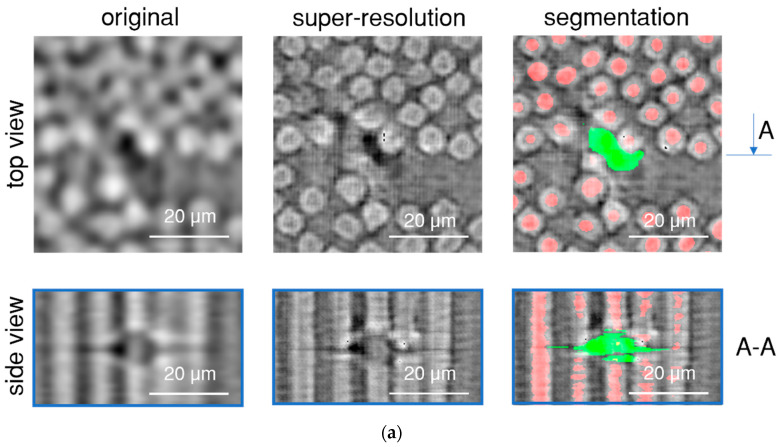

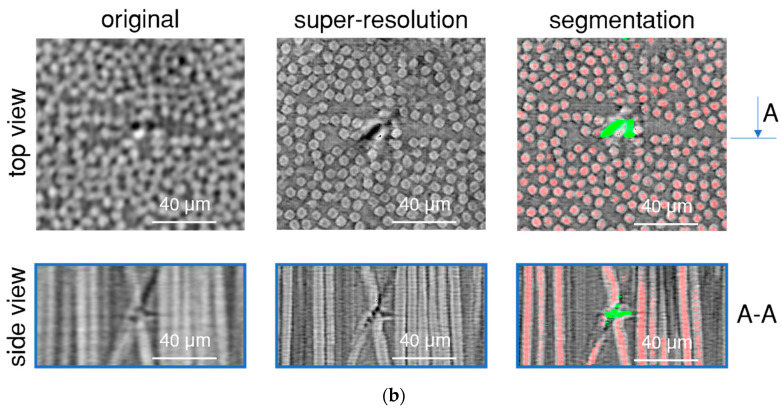
Segmentation of possible voids and fibre break identification in SR images: (**a**) example of common quality enhancement of voids; (**b**) challenging case of fibre break identification where the algorithm was not able to identify it automatically.

In low-resolution images (see Figure 5), due to the continuous fast scanning, the representation of voids consists of a few different artefacts; void vortices (only visible in 3D), the size of fibre breaks being much larger than the fibre cross-section, and voids can be superimposed by fibres, beam hardening, and grey scale inhomogeneities around voids. As these types of defects are not present in the training data, the super-resolution algorithm improves the clarity of not only the fibres and voids but also the artefacts present in the original image. This presented a challenge in segmenting the voids and accurately locating their centres for further analysis. The results of the super-resolution enhancement of voids are accurate in most cases. Figure 7a shows one such example. The void is more clearly visible without the small dark artefacts that appear between the fibres due to their tight packing. The same effect of false voids was observed in the training data. The U-Net segmentation accurately identifies the boundaries of the void, allowing us to locate its centre with sufficient accuracy to identify fibre breaks.

Figure 7b displays one of the most challenging cases of fibre break quality enhancement. In this example, the fibre break has a pronounced vortex artefact, which increased the void size in the super-resolution image. Figure 7b presents the segmentation of this dark region. Despite the artefacts, and shape and size differences, the centre of the segmented fibre break is located in the correct place due to the symmetry of the 3D vortex artefact.

Figure 5b shows the 3D visualisation of the segmented voids. The 3D visualisation shows the vortex artefacts of the voids. Unfortunately, it was not possible to train the segmentation algorithm to identify only the voids without the artefacts.

### 3.2. Fibre Break Identification

Fibre breaks are found amongst the segmented voids. After manual analysis of a few fibre breaks, an average volume was calculated, which mostly varies between 1000 and 50,000 pixels in volume and the diameter of circumscribed ellipsoids is longer than 20 pixels. After filtering according to these parameters, only voids that could potentially be fibre breaks remain. The voids with a volume much larger than the volume of the average fibre break are treated separately, as such voids may represent a cluster of fibre breaks.

Fibre break identification is performed according to the procedures described in Section 2.5, where only a central point of the fibre break is recorded (see Figure 6). Using the first method, if an initial stage image is not available, then the accuracy of the fibre break detection depends strongly on the search window and the number of slices used in the analysis. If the search parameters are taken from the statistical analysis of void and fibre size (how many slices a void occupies), then the accuracy of the fibre break identification is not as high (see Table 2). However, if the search parameters are optimised (by trial and error), the accuracy of fibre break identification will increase, but will still not be sufficient for flawless identification of all fibre breaks.

If a scan before loading is available, then the second method can be used. This method provides better results, and there is only one parameter to optimise, the minimum distance from a void centre to the fibre trajectory, in order to consider the analysed void as a fibre break. Table 2 presents the results of the fibre break identification using both methods.

The accuracy of the fibre break identification is lower when analysing volumes with a large number of fibre breaks. This is because the algorithm is not able to recognise individual fibre breaks in densely packed fused objects, as shown in Figure 5.

At this stage of image processing, it is challenging to separate such clusters into individual voids. During the image processing, such clusters could be handled manually by an operator who would be shown the low-resolution, super-resolution, and segmented images, similar to Figure 7, and the operator can decide if there is a fibre break in the images. The operator could remove all the false positive errors from the results, leaving only false negatives unidentified. Using this approach with super-resolution analysis, it is possible to identify most fibre breaks in low-resolution images of continuously loaded specimens in a reasonable amount of time.

**Figure 8:**
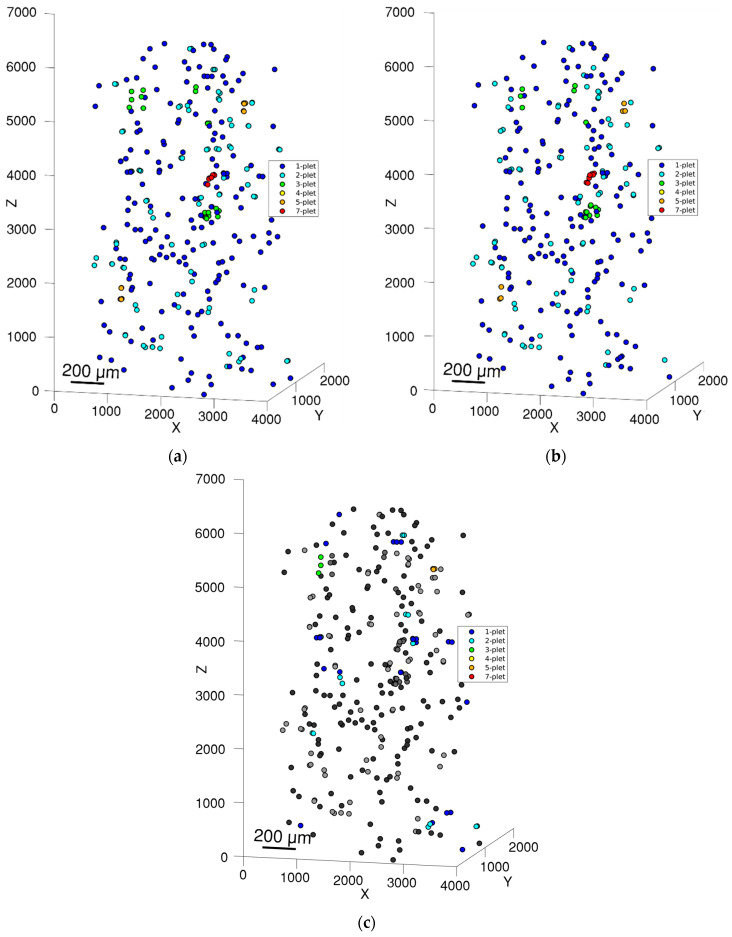
Location of all fibre breaks and their clusters for (**a**) manual inspection and (**b**) semi-automated inspection. (**c**) Small differences are highlighted by colour.

Fibre breaks were clustered using the geometric criteria described in Section 2.5: fibre break identification. For clusters, we used the results from the second method when the initial image was analysed. Table 3 summarises the results of the fibre break cluster identification, where we can see small differences in cluster identification (see Figure 8c). This is because the automated algorithm and an operator cannot locate the centre of the fibre break with the same coordinates. The average distance between manual and automatically calculated coordinates is about 4.7 µm and can be up to 10 µm for large fibre breaks or fused objects. These deviations are comparable to the radial distance of 13 µm in the geometric criteria and can affect the clustering of the fibre breaks.

Image processing of the enhanced images takes a considerable amount of time. Once the super-resolution network was trained, the SR application took about four hours to increase the resolution of the whole volume, six hours to segment an SR image into fibres and voids, and one hour for image processing, tracking fibre trajectories, calculating void sizes, and identifying fibre breaks. In addition, approximately one hour of manual work was required to analyse large objects and eliminate false positives for the image before failure (79 large objects and 328 potential fibre breaks were checked). This is much faster than the 3–5 working days required for manual analysis, especially when considering that one hour of hands-on time was required.

Super-resolution can increase the speed of identification not only of fibre breaks in unidirectional composites but also of other manufacturing defects in composite materials such as delamination, matrix cracking, voids, or porosity. The developed algorithm can help researchers working on models for strength assessment based on fibre breaks. One can try to train the algorithm on one material and transfer the super-resolution capabilities to other similar materials, such as demonstrated by training on T700SC and validating on 34-700WD. However, there are limitations to using machine learning algorithms on data that are significantly different from the training dataset; such algorithms will not be able to work with significantly distinct data. If a new type of feature is introduced in the images, machine learning algorithms will produce unexpected results and miss important features. One reason why features may be missed after SR is that, in low-resolution images, there is not enough information about them to reconstruct correctly. The spatial resolution of the LR image determines the minimum feature size that can be reconstructed, which should be at least two pixels per feature size. Another reason is that the features are not well represented in the training data, in which case the model can fill in the feature with the structure it is most familiar with [51]. For example, in this work, the neural network was unable to correctly remove vortex artefacts and improve the quality of all voids. The limits on how significantly the images can differ from each other remain a topic for further research. Furthermore, future work could be devoted to the investigation of more versatile neural networks that are trained on different materials at different resolutions.

## 4. Conclusions

A super-resolution algorithm has been designed and applied in order to improve the image quality of low-resolution synchrotron CT scans. The algorithm is based on deep learning architecture developed by combining Enhanced Super-Resolution GAN and CycleGAN. The neural networks were trained on high-resolution and low-resolution scans of a stationary carbon fibre composite and applied to another much larger low-resolution image of a continuously loaded specimen.

As a result, the super-resolution images have more precise fibre and void boundaries with only minor deep learning-based artefacts. To segment the images, we chose RootPainter software, which was the most suitable algorithm for our case. With this software, precise identification of fibres and voids was achieved. The quality of the segmentation allows the study of fibre trajectories and void locations.

Super-resolution processing enabled automated identification of fibre breaks using void location and fibre trajectories. Two algorithms have been implemented: one using the information from only the loaded image and the other using fibre trajectories from the unloaded image. The average accuracies of the methods are 76% and 82%, respectively, for the fully automated process and 86% and 92% for the semi-automated process with a miss rate of less than 5.3%. The fibre break clustering outputs similar results, with minor deviations, due to inconsistent fibre break centre locations. Super-resolution makes it possible to use faster, low-resolution in situ CT scans on continuously loaded specimens with limited compromises on the quality of physical parameter identification. The developed methodology can be used for faster, but less accurate, fibre break identification for strength assessment models.

## Figures and Tables

**Table 1:** SRCT test and scan parameters for different purposes.

Purpose	Training Set, Stationary		Validation Set, Continuous Loading
	High-Resolution	Low-Resolution	
Material	T700SC	T700SC	34-700
Sensor size (px^2^)	2560 × 2160	2560 × 2160	2016 × 1716
Sensor pixel size (µm)	6.5	6.5	11.0
Energy (kV)	15	15	20
Exposure time (ms)	250	80	9
Microscope magnification	20×	4×	10×
Voxel size (µm)	0.325	1.625	1.1
Number of projections per volume	2000	2000	1000
Propagation distance (mm)	30	100	60
Displacement rate (µm/s)	-	-	1.4–1.6
Number of volumes acquired before failure	1	1	17
Testing time per scan (s)	500	160	9

**Table 2:** Statistics of the automated fibre break identification with both methods.

	98% Load Method 1 (Stat.)	98% Load Method 1 (Opt.)	98% Load Method 2	94% Load Method 1 (Opt.)	94% Load Method 2	75% Load Method 1 (Opt.)	75% Load Method 2
Manual	299	299	299	248	248	78	78
True positive	258	266	272	225	230	74	75
False positive	70	51	33	43	25	14	6
False negative	41	33	27	23	18	4	3
Large objects	79	79	79	39	39	1	1
Breaks from large objects	18	18	18	12	12	1	1
Automatic accuracy	0.70	0.76	0.82	0.77	0.84	0.80	0.89
Semi-automatic accuracy	0.79	0.86	0.92	0.85	0.93	0.82	0.92
Miss rate (%)	8.2	5.3	3.2	4.7	2.5	3.9	2.6

**Table 3:** Number of fibre break clusters identified with the manual and automated inspection methods.

		1-Plet	2-Plet	3-Plet	4-Plet	5-Plet	7-Plet
98% load	manual	175	43	7	0	2	1
	auto	170	41	7	0	2	1
94% load	manual	145	34	6	0	2	1
	auto	147	29	6	0	2	1
75% load	manual	45	10	1	0	2	0
	auto	42	9	2	0	2	0

## Data Availability

The data used in this research will be made available upon request.
